# Electrohydrodynamic jet printing of gelatin-GPTMS-PEO hybrid biomaterial inks for high-resolution fibre patterning

**DOI:** 10.3389/fbioe.2026.1929042

**Published:** 2026-10-01

**Authors:** Sara Sadati, Marcus J. Swann, Meera Unnikrishnan, Dmitry Isakov

**Affiliations:** 1 WMG, University of Warwick, Coventry, United Kingdom; 2 Division of Biomedical Sciences, Warwick Medical School, University of Warwick, Coventry, United Kingdom; 3 5D Health Protection Group, Liverpool, United Kingdom

**Keywords:** electrohydrodynamic jet printing, gelatin, biomaterial ink, fibre patterning, fibrous biomaterial architectures, nanobiotechnology

## Abstract

Electrohydrodynamic jet printing (EHD jet printing) enables high-resolution direct writing of fibrous biomaterial architectures, which are of interest for future biointerface, scaffold-mimetic, and sensing-oriented platforms. Yet EHD jet printing of aqueous gelatin systems remains challenging because of temperature-dependent viscosity increase, capillary instability, and narrow cone-jet stability windows. Here, we adapt an aqueous gelatin-GPTMS-PEO hybrid biomaterial ink for near-field EHD jet printing using acetic acid as a process-modifying additive. Acidification improved precursor clarity and extended the low-viscosity processing window during cooling, the printable formulation had an apparent viscosity of 49.6 mPa ⋅ s at 40 °C, electrical conductivity of approximately 102 mS ${\text{m}}^{-1}$ , and surface tension of 47.2 mN ${\text{m}}^{-1}$ . Jetting behaviour was mapped across applied voltage, working distance, and flow rate, identifying dripping, pulsating, stable cone-jet, and unstable regimes. Stable fibre deposition was achieved within a process window defined by $Bo_{E}$ = 0.26–0.74, d/h≤0.50 , and Ca = 0.025–0.050. FTIR-ATR and Raman spectroscopy supported retention of the principal gelatin and PEO vibrational signatures after acidification and printing, with no major spectroscopically detectable degradation. Under optimised conditions, continuous fibres with a mean diameter of 1.19 ± 0.49 µm were obtained, and programmed parallel patterns were deposited with a mean absolute spacing error of 3.3% relative to a nominal 1 mm spacing. These results establish a process-structure platform for EHD printing of aqueous gelatin-based hybrid biomaterial inks and provide a foundation for patterned fibrous architectures in nanobiotechnology.

## 1 Introduction

Microscale and nanoscale fibrous biomaterial architectures are of importance in tissue engineering, regenerative medicine, biosensing, drug delivery, and biointerfaces because fibre diameter, porosity, alignment, and spatial organisation influence material-cell interactions and device function (Zhu et al., 2020; Keirouz et al., 2023; Wu, 2021; Parvin et al., 2025; XiangGu et al., 2016). Recent examples include nanofibre scaffolds for stem and stromal cell expansion, core–shell or layered fibre arrays for tissue regeneration, nanofibrous carriers for sensitive biological cargo, and aligned fibre patches or injectable fibrous structures for controlled microenvironment design (Iwakami et al., 2026; Mao et al., 2026; Stojanov and Berlec, 2020; Kumar et al., 2020; Wang et al., 2024). While these studies show the importance of controlled fibrous architectures in nanobiotechnology, they also highlight the need for fabrication methods that can define fibre placement as well as fibre morphology.

Electrohydrodynamic (EHD) jet printing is a high-resolution direct writing method that uses electric stresses to deform a liquid meniscus and draw a fine jet from the cone apex (Mkhize and Bhaskaran, 2022; Onses et al., 2015; Park et al., 2007). Unlike conventional electrospinning, which often relies on whipping instabilities to form random fibre mats, near-field EHD printing can deposit continuous fibres and ordered patterns when a stable cone-jet is maintained. However, translating EHD direct writing from thermoplastic systems such as melt electrowriting to aqueous, biofunctional solution-state inks remains challenging (Wu, 2021; Mueller et al., 2024; Farrugia et al., 2013).

Gelatin is an attractive biopolymer for aqueous biomaterial inks because it is derived from collagen, contains cell-adhesive peptide motifs, is enzymatically degradable, and can be chemically modified (Mohanto et al., 2023; El-Seedi et al., 2023). Direct EHD printing of gelatin-rich aqueous inks is nevertheless difficult as gelatin solutions show temperature-dependent gelation, limited solution elasticity, and formulation-dependent apparent viscosity, all of which influence cone-jet stability and fibre formation (Mkhize and Bhaskaran, 2022; Korkut et al., 2008; Park and Moon, 2006; Roldan et al., 2023; de Farias et al., 2023). Unmodified gelatin networks also tend to have limited thermal and aqueous stability, motivating chemical modification or blending approaches for processing and post-deposition integrity (Liang et al., 2025).

Polymer blending and GPTMS-assisted hybrid-network formation provide complementary routes to tune gelatin processability. Poly (ethylene oxide) (PEO) or related polyethers can increase chain entanglement and support continuous jet formation, while silane coupling agents such as (3-glycidyloxypropyl)trimethoxysilane (GPTMS) can contribute to hybrid organic–inorganic gelatin network formation through gelatin coupling and silanol/siloxane formation (Dias et al., 2017; Zhang et al., 2006; Righi et al., 2012; Monavari et al., 2024; Do et al., 2026; Zhang et al., 2023). Recently, we have developed gelatin-GPTMS-PEO hybrid systems and showed that formulations containing 2 wt% PEO could provide a useful balance of flexibility, hydrolytic stability, and functional responsiveness (Sadati et al., 2026a; Sadati et al., 2026b). However, translation of this hybrid system into high-resolution EHD direct writing has not yet been established.

Despite recent success in gelatin fibres fabrication (El-Seedi et al., 2023; Roldan et al., 2023), the main unresolved issue is the lack of process-structure mapping for aqueous gelatin hybrid inks under near-field EHD conditions. Stable patterning requires a non-pulsating cone-jet, but the operating window depends on coupled precursor properties, including apparent viscosity, thermal gelation behaviour, conductivity, surface tension, and fluid flow rate. For gelatin-GPTMS-PEO systems, these factors are further complicated by solution-state heterogeneity, temperature-sensitive gelatin association, PEO-mediated entanglement, and possible GPTMS-derived network formation, as supported by previous gelatin–GPTMS studies (Sadati et al., 2026a). A framework that links precursor modification, dimensionless jetting regimes, fibre morphology, and programmed pattern fidelity is therefore needed.

The present study addresses this gap by adapting an aqueous gelatin-GPTMS-PEO (hereafter GG-PEO) hybrid biomaterial ink for near-field EHD jet printing, and by identifying the conditions required for stable cone-jet formation, continuous fibre deposition, and programmed patterning. Acetic acid is introduced as a process-modifying additive to improve apparent precursor homogeneity, regulate apparent viscosity and cooling-induced flow behaviour, and increase electrical conductivity. Its role is interpreted in terms of solution-state interactions and processability rather than as an additional functional biomedical component. Rheological, electrical, surface, spectroscopic, and morphological analyses are combined with regime mapping across voltage, working distance, flow rate, electric Bond number, capillary number, and geometry ratio. The resulting fibre diameter distributions and patterning fidelity are then correlated with the mapped jetting regimes, establishing a physically informed processing framework for aqueous gelatin-based hybrid inks for future fibrous biointerface, scaffold mimetic, and sensing-oriented studies.

## 2 Materials and methods

### 2.1 Preparation of gelatin-GPTMS-PEO precursor solutions

GG-PEO precursor solutions were prepared as aqueous hybrid biomaterial inks for electrohydrodynamic jet printing. The formulation is described here as a gelatin-based hybrid biomaterial ink or EHD-printable biomaterial ink, rather than as a biologically validated bioink. Only the selected acidified formulation containing 6% v/v acetic acid was carried forward for detailed physicochemical characterisation and printing. This concentration was chosen as a process-modifying condition for the present EHD-printing study. Gelatin (type A, 300 bloom, 70%–90% biuret, Sigma-Aldrich) was dissolved in deionised water at 50 °C under continuous stirring to obtain a 10% w/v stock solution. After the gelatin had become visibly solubilised (3-glycidyloxypropyl)trimethoxysilane (GPTMS, ≥ 98%, purchased from Sigma-Aldrich) was added at a GPTMS:gelatin mass ratio of 1:5 (which was the optimised mass ratio in our previous work (Sadati et al., 2026a)), and the mixture was stirred for 1 h at 50 °C. GPTMS was used as a bifunctional silane network-forming modifier that can contribute to hybrid network formation through reaction of its epoxide group with nucleophilic groups on gelatin and through hydrolysis and condensation of trimethoxysilane groups to form silanol and siloxane structures (Mahony et al., 2014). In parallel, a 10% w/v poly (ethylene oxide) (PEO, Mw = 300,000, Alfa Aesar) stock solution was prepared in deionised water and stirred until visually homogeneous.

The gelatin-GPTMS precursor and PEO stock solution were then mixed together to give a final formulation containing 5% w/v gelatin, GPTMS at a 1:5 mass ratio relative to gelatin, and 2% w/v PEO. Acetic acid (AA, glacial ≥ 99.7%, Sigma-Aldrich) was added at 6% v/v as a process-modifying additive to improve apparent precursor homogeneity and support stable electrohydrodynamic jetting. The acidified mixture was stirred at 50 °C for at least 30 min before characterisation and printing. The optical transition from turbidity to transparency was interpreted as improved apparent homogeneity rather than definitive evidence of complete molecular miscibility. Two independent batches were prepared for each formulation (non-acidified and acidified). These batches were used for the rheological, electrical conductivity, and surface tension measurements. For each batch, viscosity and electrical conductivity were measured in five repeat measurements, while surface tension was measured in ten repeat measurements. The reported values represent the mean ± standard deviation calculated from these replicate measurements.

### 2.2 Rheological characterisation

Rheological measurements were performed to assess the process-relevant flow behaviour, temperature sensitivity, and cooling-induced increase in apparent viscosity of precursor solutions prepared with and without acetic acid. Viscosity measurements were performed using an IKA Rotavisc lo-vi rheometer, equipped with calibrated SP-1, and SP-2 standard spindles for measuring viscosities in the ranges of 30–200 mPa ⋅ s and SP-4 for higher viscosities, respectively, with an accuracy of 1%. Because the measurements were conducted using rotational speed sweeps rather than a fully defined shear-rate-controlled geometry, the data are reported as apparent viscosity as a function of spindle speed. For each formulation, a total volume of 200 mL was prepared to ensure complete spindle immersion during rotational measurements. Samples were equilibrated at 50 °C for 30 min before testing.

Apparent viscosity was first measured during a rotational speed sweep to identify the operational torque window of the instrument, defined as 20%–80% torque. Temperature-dependent apparent viscosity was then recorded at a fixed rotational speed of 60 rpm while the sample cooled from 50 °C to below 25 °C. The cooling transition was defined operationally as the temperature of rapid viscosity increase or flow arrest under the measurement conditions, rather than as a true rheological gel point. This cooling experiment was used to estimate the thermal processing window and should be interpreted as a bulk flowability assessment rather than a direct measurement of fibre solidification after electrohydrodynamic deposition.

The cooling behaviour was evaluated by transferring thermally equilibrated samples into a water bath maintained at 16 °C. Apparent viscosity was recorded at fixed 10 min intervals until the sample exceeded the instrument measurement range or no longer flowed under the measurement conditions. These measurements were used to compare the relative pot life of the control and acidified precursors under cooling conditions relevant to handling and printing.

### 2.3 Electrical conductivity measurements

Electrical conductivity was measured using a Keithley 2,401 SourceMeter (Keithley Instruments, United States) configured for four-wire resistance sensing to minimise lead-resistance errors. Measurements were performed using a parallel stainless-steel electrode cell with an electrode spacing of 3 cm and an effective electrode area of 6.36 ${\text{cm}}^{2}$ , giving a calculated cell constant of 0.472 ${\text{cm}}^{-1}$ . A constant current of 0.1 mA was applied with a voltage compliance limit of 1 V. The cell constant was validated using aqueous NaCl standards of known conductivity. To reduce electrode-polarisation effects, resistance was measured under forward and reverse current polarities, and the average resistance was used. Conductivity measurements were performed at 40 °C on 6% (v/v) acetic acid, the non-acidified GG-PEO precursor, and the acidified GG-PEO + AA precursor to match the EHD printing temperature. Electrical conductivity, σ , was calculated from

$$\sigma =\frac{K_{\text{cell}}}{R},$$

where $K_{\text{cell}}$ is the electrode cell constant and R is the measured resistance. The resulting conductivity values were used to calculate the electrohydrodynamic scaling parameters discussed in the Results section.

### 2.4 Surface tension measurements

Surface tension was measured at 40 °C using the pendant drop method with a custom microscopic imaging system. Precursor solutions were equilibrated to the printing temperature before measurement. Droplets were formed using a 1 mL syringe fitted with a 0.5 mm diameter nozzle, matching the nozzle geometry used for EHD printing. The imaging system was calibrated using deionised water as a reference fluid. For each formulation, at least ten separate droplets were generated, allowed to reach a stable pear-shaped profile, and imaged. The droplet variation reflects within-formulation measurement variability for each prepared solution. Droplet profiles were analysed using the Pendant Drop plugin in ImageJ and fitted using the Young–Laplace equation. Surface tension, γ , was calculated as

$$\gamma =\Delta \rho gL^{2},$$

where Δρ is the density difference between the liquid and air, g is gravitational acceleration, and L is the characteristic length obtained from profile fitting.

### 2.5 Electrohydrodynamic jet printing

Electrohydrodynamic jet printing was performed using a custom-built near-field printing system. The system comprised a high-voltage power supply (Keithley 2290E-5), a custom stepper-motor-driven syringe pump with syringe heating, a Physik Instrumente U-723 PILine XY stage, a Physik Instrumente L-306 Z stage, and a Thorlabs DCU223M CCD camera for meniscus monitoring. The U-723 XY stage provides a 20-nm accuracy and enables programmed axial translation at collector speeds v up to 150 mm ${\text{s}}^{-1}$ .

The syringe-pump actuation and heating control were implemented using an NI cRIO-9040 FPGA controller coupled to stepper-motor driver, thermocouple module, and digital input/output module for PID modulation required for heater control. Hardware control, parameter logging, real-time meniscus monitoring, and programmed two-dimensional toolpath generation were implemented through a centralised custom user interface developed in MATLAB App Designer (Figure 1).

**FIGURE 1:**
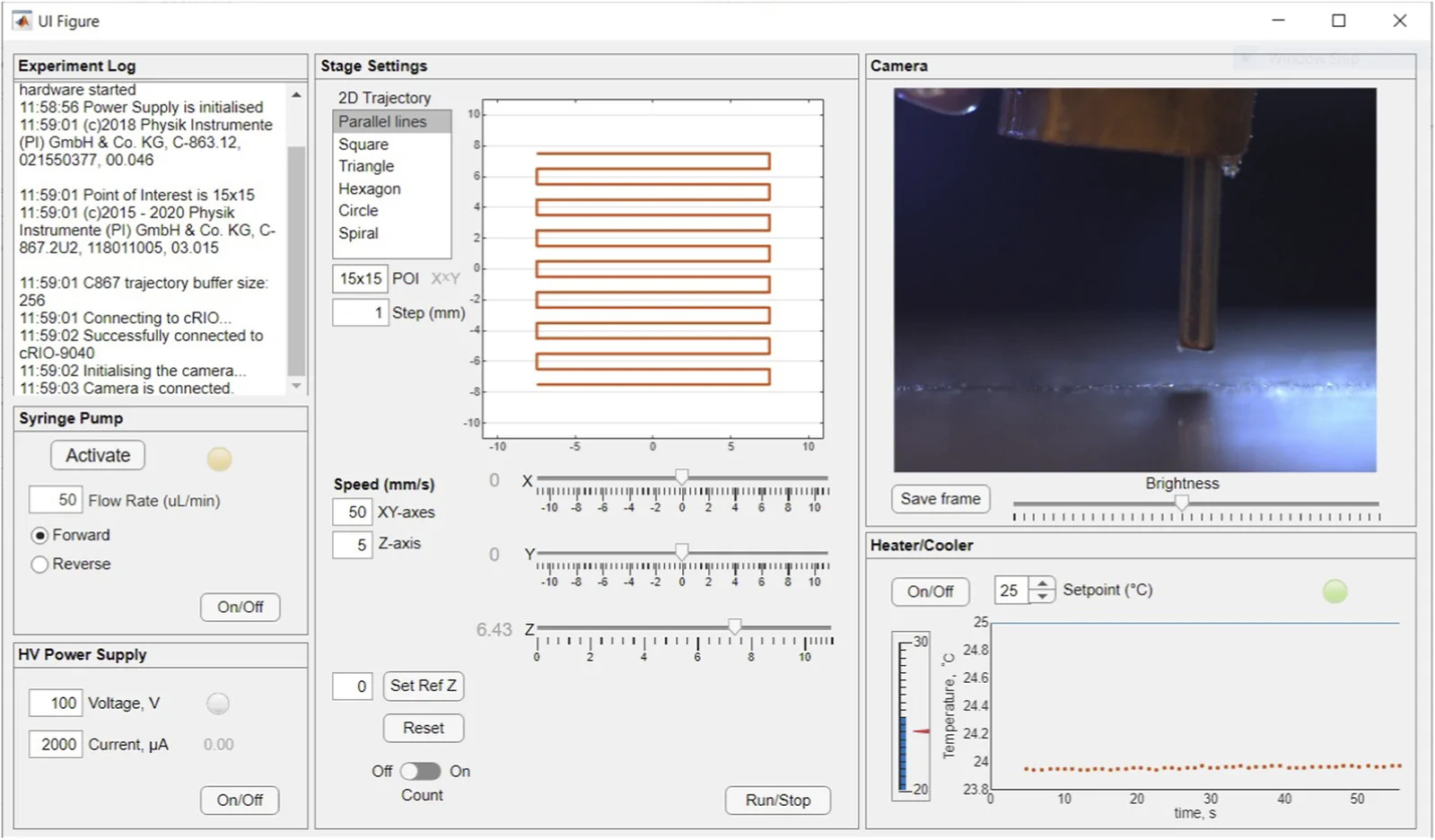
A screenshot of the front panel of the custom graphical user interface developed in MATLAB App Designer for controlling the near-field EHD printing system and logging process parameters. The interface enables real-time meniscus monitoring and user-defined control of voltage, flow rate, temperature, working distance, and programmed two-dimensional patterns with variable size and spacing.

The acidified GG-PEO + AA precursor was loaded into a syringe and maintained at 40 °C during printing to keep the formulation within the processable low-viscosity state identified by rheological characterisation. A positive voltage was applied to the needle, while the collector was electrically grounded. The applied voltage, needle-collector working distance, volumetric flow rate, collector translation speed (v) , and temperature set point were controlled through the custom interface. Printing was performed under ambient room conditions; relative humidity was not specifically measured or controlled during these experiments.

Printing conditions were screened by varying the applied voltage from 1800 to 2800 V, the working distance (h) from 0.5 to 1.5 mm, and the volumetric flow rate (Q) from 0.05 to 0.3 µL ${\text{min}}^{-1}$ . Jetting behaviour was monitored in real time using the CCD camera focused on the needle tip and meniscus region. The regime map represents independent process observations during parameter screening and is reported as a process map for the selected formulation. Four operational regimes were identified from the observed meniscus and jet dynamics: dripping, pulsating jetting, stable cone-jet printing, and unstable jetting. Under the near-field conditions used here, unstable jetting was assigned to irregular cone oscillation, intermittent jet displacement, multi-jetting, or loss of controlled deposition, rather than classical far-field whipping.

For regime mapping, the nominal electric field was estimated as E=V/h , where V is the applied voltage and h is the needle-collector distance. The electric Bond number was calculated as

$$Bo_{E}=\frac{\varepsilon_{0}E^{2}d}{\gamma },$$

where $\varepsilon_{0}$ is the vacuum permittivity, d is the needle inner diameter, and γ is the measured surface tension of the precursor. The capillary number was estimated as

$$Ca=\frac{\eta U}{\gamma },$$

where η is the process-relevant apparent viscosity and U is the mean exit velocity calculated from U=Q/A , with A being the needle cross-sectional area. These dimensionless groups were used as scaling parameters to compare operating conditions, not as exact universal predictors of cone-jet onset. Because both Oh and Ca scale linearly with η , uncertainty in the apparent-viscosity value propagates proportionally into these dimensionless numbers; they are therefore interpreted as approximate, process-specific descriptors.

### 2.6 FTIR-ATR and Raman spectroscopy

Fourier transform infrared spectroscopy in attenuated total reflectance mode (FTIR-ATR) and Raman spectroscopy were used to assess whether the principal gelatin and PEO vibrational signatures remained detectable after acidification and electrohydrodynamic jet printing. The measurements were interpreted as spectroscopic evidence of retention of the main molecular signatures rather than as a quantitative assessment of crosslink density, GPTMS conversion, or PEO crystallinity.

FTIR-ATR spectroscopy was performed over a range of 4,000–500 cm^-1^ with 4 cm^-1^ resolution and 32 scans using a Bruker Tensor 27 FTIR spectrometer. EHD-printed fibres were deposited on glass slides because conductive substrates such as platinum-coated wafers caused electrical short-circuiting during printing. The glass substrate was therefore used as a practical insulating collector, although its contribution to the Si–O–Si stretching region was considered during spectral interpretation. Spectra from printed fibres were compared with reference spectra collected from solvent-cast gelatin, gelatin–GPTMS film, PEO, blended GG-PEO films, and the bare-glass substrate. Spectra were acquired over the mid-infrared region and used to identify characteristic gelatin amide bands, PEO ether vibrations, and possible GPTMS-related siloxane features, where detectable.

Raman spectroscopy was performed as a complementary vibrational technique for molecular characterisation of the EHD-printed fibres and reference films. Reference spectra were acquired from solvent-cast gelatin, gelatin–GPTMS film, PEO, and blended films to establish the principal vibrational features of each component. Printed fibre spectra were then analysed to evaluate whether acid modification and EHD processing produced major spectroscopically detectable changes. Measurements were performed using a confocal Raman microscope with 442 nm excitation. Laser power was adjusted according to sample scattering response, with lower power used for PEO-containing samples to minimise thermal damage. Spectra were acquired across the fingerprint region, 400–1800 ${\text{cm}}^{-1}$ , and the C–H stretching region, 2,800–3,100 ${\text{cm}}^{-1}$ , followed by baseline correction and spectral processing.

### 2.7 Field-emission scanning electron microscopy

The morphology of EHD-printed fibres and patterned structures was examined using field-emission scanning electron microscopy (FE-SEM; TESCAN CLARA). Fibres were printed onto glass slides, mounted on aluminium stubs using conductive carbon tape, and sputter-coated with gold for 60 s before imaging. FE-SEM imaging was performed at an accelerating voltage of 5 kV. Images were acquired from multiple regions of each printed sample to assess fibre continuity, beading, coiling, surface morphology, and fibre diameter distributions.

Fibre diameters were measured from calibrated FE-SEM images using ImageJ. For each printing condition, at least 30 fibres or fibre segments were selected from different image regions where the fibre edges could be resolved clearly. Diameter measurements were taken perpendicular to the fibre axis. Where repeated measurements were made on the same fibre or image region, the values were averaged before statistical analysis to avoid over-weighting a single local feature. Measurements of fibre diameter and spacing were obtained from calibrated imaging fields across three independent images, using representative printed samples prepared on separate days.

### 2.8 Data analysis

Image analysis was performed using ImageJ software (National Institutes of Health, United States) (Schneider et al., 2012) on spatially calibrated optical or FE-SEM images. Fibre diameter distributions were obtained from a minimum of 30 measurements per printing condition. Inter-fibre spacing in programmed parallel-line patterns was measured from calibrated low-magnification images by determining the centre-to-centre distance between neighbouring printed lines. Patterning fidelity was reported as the measured spacing relative to the programmed spacing and as the mean absolute spacing error.

Graphical presentation of material characterisation data was performed using DataGraph (Visual Data Tools, Chapel Hill, NC, USA). Quantitative results are reported as mean ± standard deviation. Fibre diameter distributions are reported descriptively from fibre image measurements. Dimensionless parameter calculations, jetting-regime maps, and fibre-diameter distribution plots were generated using custom Python scripts with NumPy and Matplotlib.

## 3 Results

### 3.1 Solution properties and effect of acetic acid modification

The visual appearance of the precursor solutions provided an initial indication that acetic acid modified the solution state of the GG-PEO system (Figure 2a). The non-acidified precursor appeared turbid, indicating mesoscale heterogeneity in the aqueous polymer mixture (Tao et al., 2023). According to Flory-Huggins polymer-blend theory, miscibility is determined by the interplay between the entropy of mixing and the enthalpic interaction parameter χ (Rudin and Choi, 2013; Manias and Utracki, 2014). For high-molecular-weight polymers, the entropic contribution to mixing is small, meaning that even moderately unfavourable enthalpic interactions can promote phase separation or polymer-rich domain formation. In the present gelatin-PEO aqueous hybrid system, the observed turbidity is therefore consistent with insufficiently favourable gelatin-PEO interactions and local network heterogeneity. However, visual turbidity alone does not allow χ to be quantified or a single phase-separation mechanism to be assigned.

**FIGURE 2:**
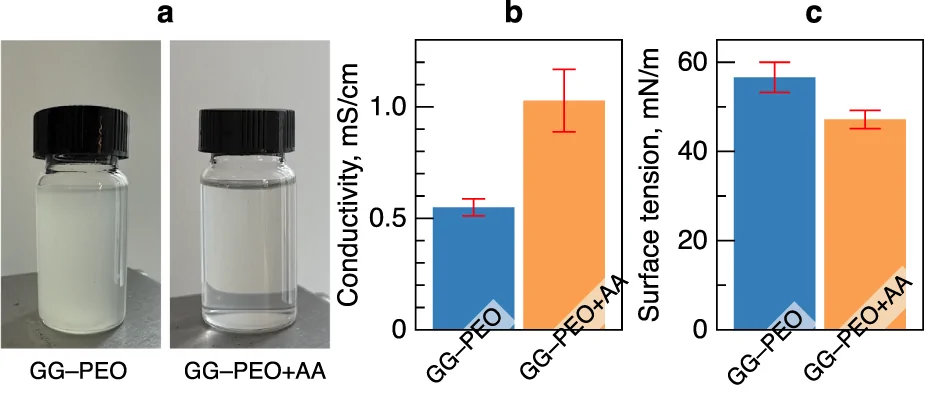
Physicochemical effect of acetic acid on gelatin-GPTMS-PEO precursor solutions. **(a)** Visual appearance of the non-acidified precursor (GG-PEO) and the acetic-acid-modified precursor (GG-PEO + AA), showing a transition from turbidity to optical transparency. **(b)** Electrical conductivity and **(c)** surface tension for the tested solutions/formulations. Data are presented as mean ± standard deviation from repeated measurements within the prepared formulations.

Addition of acetic acid at 6% v/v produced an optically transparent precursor (Figure 2a). This transition is interpreted as improved apparent homogeneity rather than definitive evidence of complete molecular miscibility. The measured pH = 2.6 with acid, and pH = 5.8 without acid, confirmed that the precursor was acidified after acetic acid addition. Therefore, the observed change in appearance was likely due to the protonation of gelatin amine groups that led to increased electrostatic repulsion between gelatin chains. Consequently, transient gelatin-gelatin associations and gelatin-water, gelatin-PEO, and PEO-water interactions were altered. Acetic acid may influence GPTMS hydrolysis and condensation kinetics in solution; however, the FTIR-ATR spectra of acidified and non-acidified GG-PEO films did not show major changes in the principal gelatin and PEO bands, as shown later in Section 3.3. This suggests that acidification did not produce major spectroscopically detectable degradation after film formation, although GPTMS conversion, siloxane formation, and solution-state hydrolysis and condensation kinetics were not quantified directly.

Electrical conductivity and surface tension were then measured because both parameters strongly influence EHD jet formation and directly reflect the physicochemical effect of acetic acid addition. The acidified precursor showed a conductivity of approximately 102 mS m^−1^ (Figure 2b), which is higher than values typically reported for EHD inks based on organic solvents, however, consistent with the ionic character of the acetic-acid modified aqueous formulation (Mkhize and Bhaskaran, 2022; Ramon et al., 2024). Using the measured conductivity and assuming a water-rich relative permittivity of $\varepsilon_{r}\approx 74$ , the charge relaxation time can be estimated as

$$\tau_{q}=\frac{\varepsilon_{0}\varepsilon_{r}}{K},$$

where $\varepsilon_{0}$ is the vacuum permittivity, $\varepsilon_{r}$ is the assumed relative permittivity, and K is the electrical conductivity. This gives an order-of-magnitude value of approximately 6 ns. The estimate should be used carefully because the dielectric constant of the GG-PEO + AA precursor was not measured directly and may change during solvent evaporation. Even allowing for uncertainty in the dielectric constant, the estimated $\tau_{q}$ is much shorter than the hydrodynamic timescales associated with cone deformation and fibre deposition. Within the assumptions of a water-rich leaky-dielectric approximation, this short relaxation time suggests that charge conduction is likely to dominate over charge accumulation on the hydrodynamic timescale of cone deformation and jet elongation. The acidified precursor can therefore be considered a highly conductive EHD ink under the conditions studied.

The surface tension of the acidified precursor was 47.2 mN ${\text{m}}^{-1}$ (Figure 2c), lower than that of pure water. This reduction is consistent with the presence of acetic acid and polymeric components at the air-liquid interface, although the individual contributions of gelatin, PEO, GPTMS-derived species, and acetic acid cannot be separated from the present measurements. Together, the visual observations, conductivity, and surface tension data show that acetic acid strongly modifies the precursor properties that define electrostatic forcing, capillary resistance, and charge transport during EHD printing.

The apparent viscosity data further supported an acid-mediated change in solution-state organisation. The control precursor showed a stronger dependence of apparent viscosity on rotational speed (Figure 3a), consistent with a structured or heterogeneous polymer solution. Since the measurement was performed as a rotational speed sweep rather than a fully defined shear-rate sweep, the results are interpreted as apparent viscosity as a function of spindle speed. After acidification, the apparent viscosity became less dependent on rotational speed over the measured range. This behaviour suggests partial disruption of transient solution-state associations and improved dispersion of the precursor components, rather than proving ideal Newtonian flow.

**FIGURE 3:**
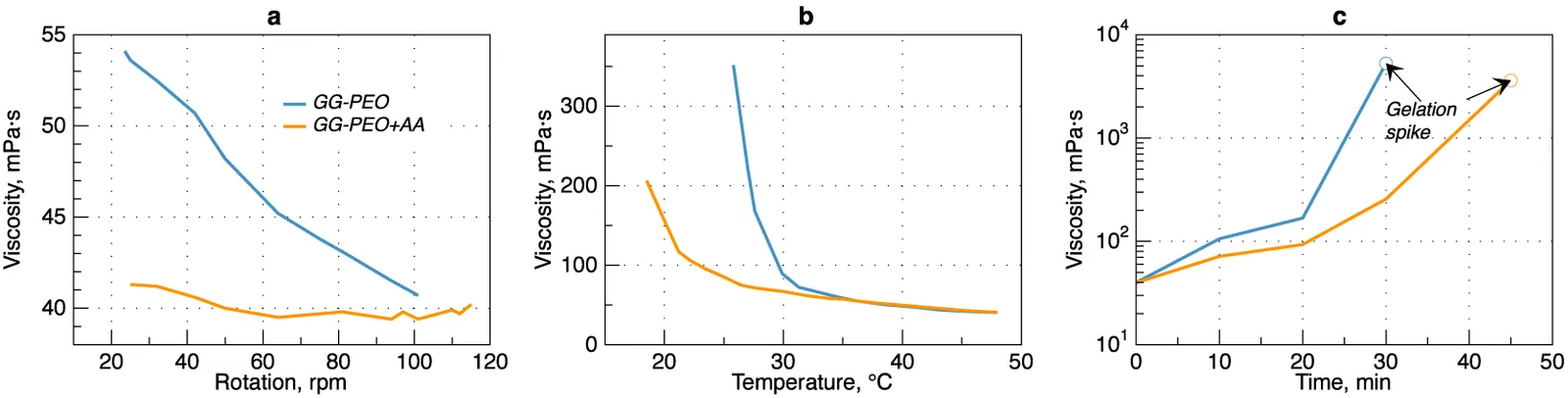
Process-relevant rheological and thermal behaviour of gelatin-GPTMS-PEO precursor solutions. **(a)** Apparent viscosity as a function of rotational speed at 50 °C for the non-acidified precursor (GG-PEO) and the acetic-acid-modified precursor (GG-PEO + AA). Here, GG denotes gelatin–GPTMS. **(b)** Temperature-dependent apparent viscosity during cooling, showing an operational rapid viscosity increase transition near 28 °C for GG-PEO and near 18 °C for GG-PEO + AA. **(c)** Time-dependent apparent viscosity during rapid cooling at approximately 16 °C, plotted on a logarithmic y-axis, indicating delayed viscosity increase and extended processable time for the acidified precursor.

The temperature-dependent apparent viscosity profiles showed that both precursors were thermally sensitive during cooling (Figure 3b). The non-acidified precursor showed a sharper increase in apparent viscosity at approximately 28 °C, while the acidified formulation remained processable to lower temperature, with the operational rapid-viscosity-increase transition shifted to approximately 18 °C. To maintain the solution in a processable, low-viscosity state during printing, the system was held at 40 °C throughout EHD operations using the integrated heating system. Under these conditions, the acidified formulation had an apparent viscosity of 49.6 ± 0.3 mPa ⋅ s. This value represents a compromise between maintaining jet continuity through polymer-chain entanglement and avoiding excessive viscous resistance to cone-jet initiation (Tian et al., 2026).

Rapid cooling experiments at approximately 16 °C were used to compare cooling-induced viscosity increase and handling time (Figure 3c). The acidified precursor showed a delayed rise in apparent viscosity relative to the control formulation, indicating an extended processable time window under cooling conditions. This test provides a process-relevant indication of thermal handling stability and pot life, but it does not establish the true gelation point of the material, because oscillatory rheology was not performed. Together, the measured apparent viscosity, conductivity, and surface tension define the balance between viscous resistance, capillary pressure, electric stress, and charge transport during cone-jet formation. The relative importance of viscous and capillary-inertial effects was estimated using the Ohnesorge number,

$$Oh=\frac{\eta }{\sqrt{\rho \gamma d}},$$

where η is the process-relevant apparent viscosity, ρ is density, γ is surface tension, and d is the nozzle diameter. Using the measured viscosity and surface tension, a precursor density of 1.10 g ${\text{cm}}^{-3}$ , and a nozzle diameter of 500 μm, the precursor gave Oh≈0.31 . As this estimate uses apparent viscosity, Oh is interpreted as a process-specific scaling descriptor. This value means that the formulation in a transitional regime where both viscous and capillary-inertial effects are relevant. Practically, this indicates that the precursor lies in a favourable processing range for stable meniscus and jet control: it is not so viscous that pumping and cone-jet initiation are strongly impeded, but not so low in viscous resistance that capillary breakup dominates uncontrollably (Ewaldz et al., 2022).

### 3.2 Electrohydrodynamic jetting behaviour and regime mapping

Stable cone-jet formation in EHD printing requires a balance between electric stress, capillary pressure, viscous resistance, and flow rate (Tian et al., 2026). To establish the processing window for the GG-PEO precursor, jetting behaviour was mapped at 40 °C over applied voltages of 1800–2800 V, working distances of 0.5–1.5 mm, and flow rates of 0.05–0.3 µL ${\text{min}}^{-1}$ . These conditions corresponded to nominal electric fields of approximately 1.2–5.6 kV ${\text{mm}}^{-1}$ . Voltages above 3000 V produced unstable jetting across the working distances examined and were therefore not included in the systematic map.

Four regimes were identified from real-time meniscus imaging: dripping/elongating, pulsating jetting, stable cone-jet printing, and unstable jetting (Figure 4a). In the dripping regime, the electric stress was insufficient to maintain a Taylor cone and the meniscus formed a pendant droplet that elongated or detached intermittently. The pulsating regime was characterised by periodic cone formation and collapse, with intermittent fibre or droplet ejection. The stable cone-jet regime showed a well-defined conical meniscus with a continuous fine jet emitted from the cone apex. Unstable jetting appeared as irregular cone oscillation, jet displacement, multi-jetting, or loss of controlled deposition. Classical whipping was not observed, which is consistent with the millimetre-scale working distances used in this near-field configuration. In contrast to far-field electrospinning, where bending or whipping instabilities can develop over longer jet flight paths, near-field EHD printing restricts the available path length and favours direct-write deposition before large bending perturbations can grow (Shin et al., 2018).

**FIGURE 4:**
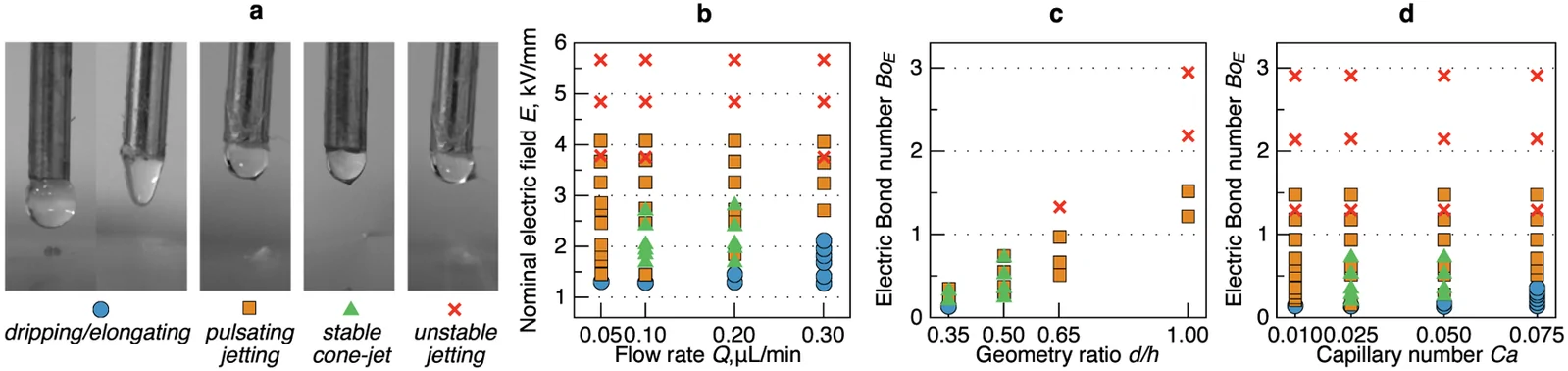
Electrohydrodynamic jetting regime map for the gelatin-GPTMS-PEO precursor at 40 °C. **(a)** Representative meniscus images showing the four observed operating regimes: dripping, pulsating jetting, stable cone-jet, and unstable jetting. **(b)** Regime map plotted as nominal electric field E=V/h versus flow rate Q . **(c)** Electric Bond number $Bo_{E}$ versus geometry ratio d/h , showing the influence of working distance relative to nozzle diameter on cone-jet stability. **(d)** Electric Bond number versus capillary number Ca , showing the combined effect of electric stress and ink flow rate. Symbols denote dripping (blue ◯ ), pulsating jetting (orange □ ), stable cone-jet (green △ ), and unstable jetting (red × ). The stable cone-jet window was confined to approximately $Bo_{E}=0.26$ –0.74, Ca=0.025 –0.050, and d/h≤0.50 under the conditions tested. Applied voltages in the range of 1800–2800 V, working distances of 0.5–1.5 mm, and flow rates of 0.05–0.3 µL ${\text{min}}^{-1}$ were investigated. Dimensionless parameters are used as process-specific scaling descriptors rather than universal stability limits.

The regime map showed that dripping occurred at low electric stress, typically at nominal electric fields below approximately 2.0 kV ${\text{mm}}^{-1}$ or electric Bond numbers below approximately 0.35 (Figures 4b,c). Dripping or microdripping was also observed at the highest flow rate tested, Q=0.3 µL ${\text{min}}^{-1}$ , even when the applied voltage was increased. This indicates that the supplied liquid volume exceeded the electrostatic stretching and removal capacity of the meniscus under the tested near-field conditions.

Pulsating jetting occupied the broadest region of the parameter space. This regime occurred during jet initiation and under conditions where charge accumulation and electrostatic deformation were sufficient to produce transient cone formation but insufficient to sustain a steady jet (Morad et al., 2016; Cheng et al., 2022). Pulsating was especially common at small working distances, where the ratio d/h was high and the electric field was strongly confined near the nozzle, and at the lowest flow rates, where low ink delivery rate reduced the duration of sustained jet emission. This behaviour is consistent with the known hysteresis of EHD cone-jet formation, where the electric field required to initiate jetting can be higher than that required to maintain an established cone-jet (Cheng et al., 2022; Shin et al., 2018).

Stable cone-jet printing was obtained only within a comparatively narrow processing window. Stable operation was observed for nominal electric fields of approximately 1.8–2.8 kV ${\text{mm}}^{-1}$ at flow rates of 0.05–0.2 µL ${\text{min}}^{-1}$ , with no stable cone-jet observed at Q=0.3 µL ${\text{min}}^{-1}$ . In dimensionless terms, the practical stable window corresponded approximately to $Bo_{E}$ = 0.26–0.74, d/h≤0.50 , and Ca = 0.025–0.050 for the conditions used for fibre deposition. These values should be interpreted as process-specific scaling bounds rather than universal stability limits, because the local field depends on needle geometry, meniscus shape, solution evaporation, and collector configuration.

The geometry ratio d/h was also a determinant of stability (Figure 4c). Stable cone-jet formation was only observed for d/h≤0.50 , corresponding to working distances of at least 1.0 mm for the 500 µm inner-diameter needle. At d/h=0.67 and d/h=1.0 , the short working distance promoted pulsating or unstable behaviour even at electric Bond numbers that supported stable jetting at larger distances. This suggests that excessively confined fields destabilised the Taylor cone, making the shortest working distances useful for jet initiation but unsuitable for sustained fibre deposition.

The capillary-number Ca representation (Figure 4d) showed that regime transitions were determined primarily by electric stress and geometry within the tested flow-rate range. Flow rate nevertheless defined the lower and upper bounds of printability. At low flow rate, limited ink delivery favoured intermittent jetting or less continuous deposition, whereas at high flow rate, the meniscus accumulated liquid faster than it could be stretched into a stable jet. Based on the combined regime map, fibre deposition experiments were performed mainly within the stable operating range using 2000–2400 V, working distances of 1.0–1.45 mm, and flow rates of 0.05–0.2 µL ${\text{min}}^{-1}$ . The 2800 V condition was also examined during SEM diameter analysis as an upper-boundary operating point to assess the onset of diameter-distribution broadening near the transition from stable to less controlled deposition. Within the stable range, Q = 0.1–0.2 µL ${\text{min}}^{-1}$ provided the most reliable continuous fibre formation and was therefore used for controlled morphology and patterning experiments.

### 3.3 Chemical characterisation of EHD-printed fibres

FTIR-ATR and Raman spectroscopy were used to evaluate whether the principal molecular signatures of the gelatin-GPTMS-PEO precursor were retained after acid modification and EHD printing (Figure 5). The spectra were compared with reference films of PEO, gelatin–GPTMS, solvent-cast GG-PEO formulations, and the glass substrate. Gelatin–PEO films reported previously (Sadati et al., 2026a) provide additional data for gelatin–PEO vibrational features, although direct comparison with the present printed fibres is limited by differences in sample morphology and formulation. FTIR-ATR spectra of the non-acidified GG-PEO film, the acidified GG-PEO + AA film, and the acidified EHD-printed fibres all showed the main bands associated with gelatin and PEO (Figure 5a). The gelatin contribution was identified from the broad N–H/O–H stretching envelope near 3,300 ${\text{cm}}^{-1}$ and the characteristic amide bands, including amide I near 1,630 ${\text{cm}}^{-1}$ , amide II near 1,540 ${\text{cm}}^{-1}$ , and amide III near 1,240 ${\text{cm}}^{-1}$ (Barth, 2007; Cebi et al., 2016). The PEO contribution was identified from the C–O–C stretching band near 1,100 ${\text{cm}}^{-1}$ and C–H stretching features near 2,930 ${\text{cm}}^{-1}$ (Pucić and Jurkin, 2012; Padmaja and Jayakumar, 2018). The retention of these bands in the printed fibres indicates that the main gelatin and PEO signatures remained spectroscopically detectable after EHD processing.

**FIGURE 5:**
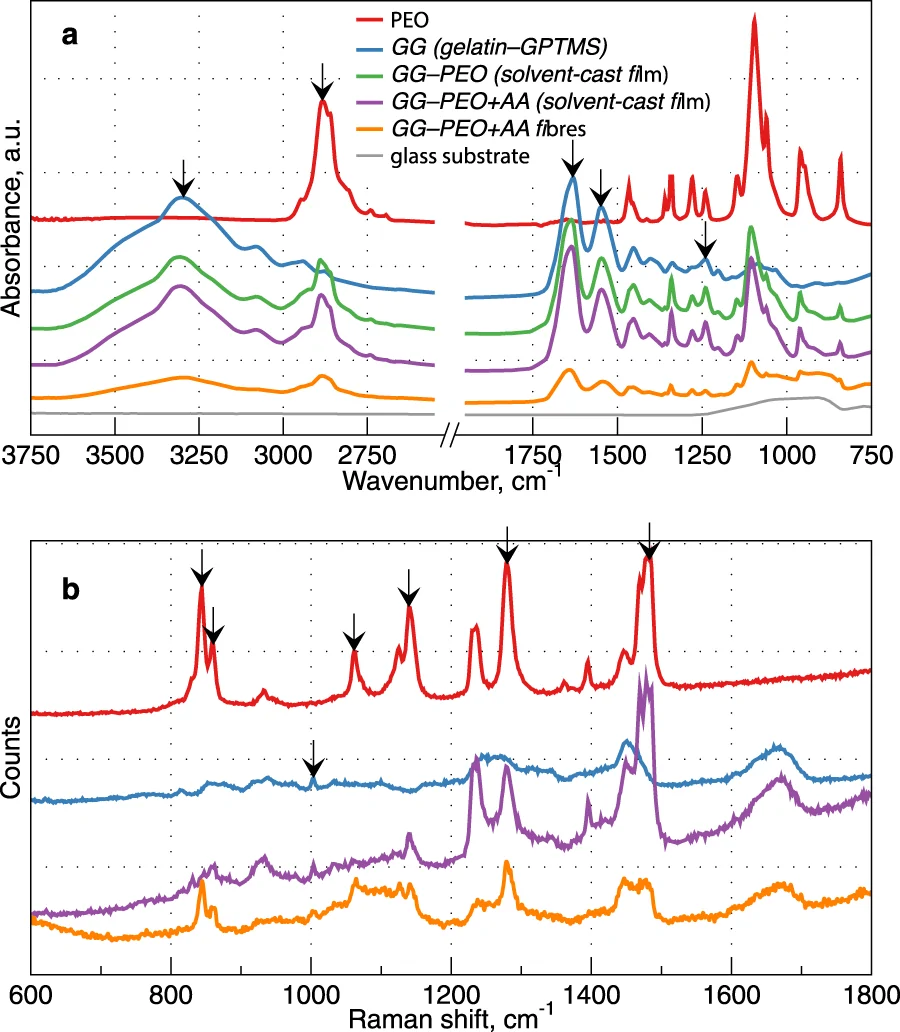
FTIR–ATR and Raman characterisation of EHD-printed gelatin-GPTMS-PEO fibres and reference materials. **(a)** FTIR–ATR spectra of EHD-printed fibres (orange), non-acidified GG–PEO film (green), acidified GG–PEO + AA film (violet), pure PEO film (red), gelatin–GPTMS film (blue), and the glass substrate. Here, GG denotes gelatin–GPTMS. Arrows indicate the principal gelatin amide bands, amide I ( ≈ 1,630 ${\text{cm}}^{-1}$ ), amide II ( ≈ 1,540 ${\text{cm}}^{-1}$ ), and amide III ( ≈ 1,240 ${\text{cm}}^{-1}$ ), together with the PEO C–O–C stretching band ( ≈ 1,100 ${\text{cm}}^{-1}$ ). **(b)** Raman spectra of the same material series, with arrows indicating characteristic PEO and gelatin vibrational modes.

Comparison of the acidified and non-acidified films did not reveal substantial shifts in the principal gelatin or PEO band positions. Similarly, the printed fibre spectrum was broadly consistent with the acidified precursor film. The lower overall FTIR absorbance of the printed fibre sample is attributed primarily to the small material volume and incomplete contact between the sparse fibre deposit and the ATR crystal, rather than to a clear compositional difference. No major new absorption bands were evident in the 1700–1800 ${\text{cm}}^{-1}$ region, where additional carbonyl-containing degradation products might be expected to contribute. Within the limits of FTIR-ATR, these observations suggest that acidification and printing did not cause major spectroscopically detectable degradation of the gelatin-PEO system.

A feature near 1,000 ${\text{cm}}^{-1}$ was more prominent in the printed fibre spectrum than in the film references. This contribution is more likely associated with the glass substrate beneath the sparse fibre deposit, rather than with a new material-specific band. This interpretation is consistent with the low areal coverage of the printed fibres and the sensitivity of vibrational spectra to substrate contributions when analysing sparse deposits. Consequently, the 1,000–1,100 ${\text{cm}}^{-1}$ region should not be used alone to assign Si–O–Si formation, because PEO C–O–C vibrations and glass-substrate overlap in this range.

Raman spectroscopy provided complementary evidence for retention of the principal polymer signatures (Figure 5b) (Taylor and Donnelly, 2020). FTIR-ATR probes vibrational transitions associated with changes in dipole moment, whereas Raman spectroscopy detects vibrations that change molecular polarisability. Raman analysis is therefore particularly sensitive to symmetric stretching modes and is less affected by water absorption than FTIR-ATR, making it useful for polymer systems with overlapping infrared bands and for spatially localised analysis of fine EHD-printed fibres. The PEO reference showed characteristic Raman features near 840 and 860 ${\text{cm}}^{-1}$ , assigned to C–C–O stretching and ${\text{CH}}_{2}$ rocking modes associated with ordered PEO domains, together with C–O–C stretching bands near 1,060 and 1,100 ${\text{cm}}^{-1}$ and ${\text{CH}}_{2}$ deformation modes near 1,280–1,470 ${\text{cm}}^{-1}$ . The gelatin reference displayed bands associated with the polypeptide backbone, including phenylalanine ring breathing and C–C stretching near 1,000 ${\text{cm}}^{-1}$ , amide III near 1,240 ${\text{cm}}^{-1}$ , proline-related ${\text{CH}}_{2}$ wagging near 1,320 ${\text{cm}}^{-1}$ , and a broad amide I band near 1,660 ${\text{cm}}^{-1}$ (Larkin, 2018; Maxfield and Shepherd, 1975).

The acidified GG-PEO + AA film retained Raman contributions from both gelatin and PEO, indicating that the principal component signatures remained present after acid modification. The Raman spectrum of the EHD-printed fibres, despite the lower signal intensity, shows that bands attributable to gelatin and PEO remained detectable in the printed fibres. The PEO-associated doublet near 840/860 ${\text{cm}}^{-1}$ appeared broader and less resolved in the printed fibres than in the pure PEO film. This broadening is consistent with reduced chain ordering or suppressed PEO crystallisation during rapid jet thinning and drying, although this interpretation cannot be quantified without complementary thermal or diffraction measurements such as DSC or XRD.

Taken together, the FTIR-ATR and Raman results support retention of the principal gelatin and PEO vibrational signatures after acidification and EHD printing. The spectra did not show evidence of major spectroscopically detectable degradation under the processing conditions identified in Section 3.2. The accompanying changes in precursor rheology are therefore more consistently interpreted as arising from acid-mediated changes in solution-state interactions and apparent homogeneity, rather than from extensive chemical transformation of the polymer components. This chemical characterisation provides the basis for interpreting the fibre morphology and diameter distributions described in the following section.

### 3.4 Fibre morphology and diameter analysis

The morphology, diameter, and spatial placement of EHD-printed fibres were characterised by FE-SEM under conditions selected from the jetting map in Section 3.2. The objective was to identify whether fibres could be formed and the jet could be translated into controlled deposition along programmed toolpaths. Three related aspects were therefore examined: (i) the morphology produced by different fluid flow and collector translation speed (v) conditions, (ii) the voltage-dependent fibre diameter distribution within the stable deposition regime, and (iii) the fidelity of programmed parallel line patterns.

#### 3.4.1 Deposition morphology under different flow-rate and collector-speed conditions

Figure 6 shows that the printed morphology was determined by ink flow rate and the relative motion between the jet and collector. At Q=0.05 µL ${\text{min}}^{-1}$ and v=25 mm ${\text{s}}^{-1}$ , fibre deposition was possible, and the deposited material generally followed the needle toolpath direction (Figure 6a). However, the tracks were discontinuous and appeared as broken or dash-like fibre segments rather than straight continuous fibres. This morphology is consistent with operation near the lower flow-rate boundary of the deposition window, where small perturbations moved the meniscus between stable emission and intermittent or pulsating jetting. Under these conditions, insufficient ink delivery promotes periodic thinning, local jet interruption, droplet ejection, and partial collapse of the deposited filament. Although these features are undesirable for precision printing, the common alignment of the deposits along the programmed direction indicates that the jet remained partially guided by the near-field electric field and substrate motion.

**FIGURE 6:**
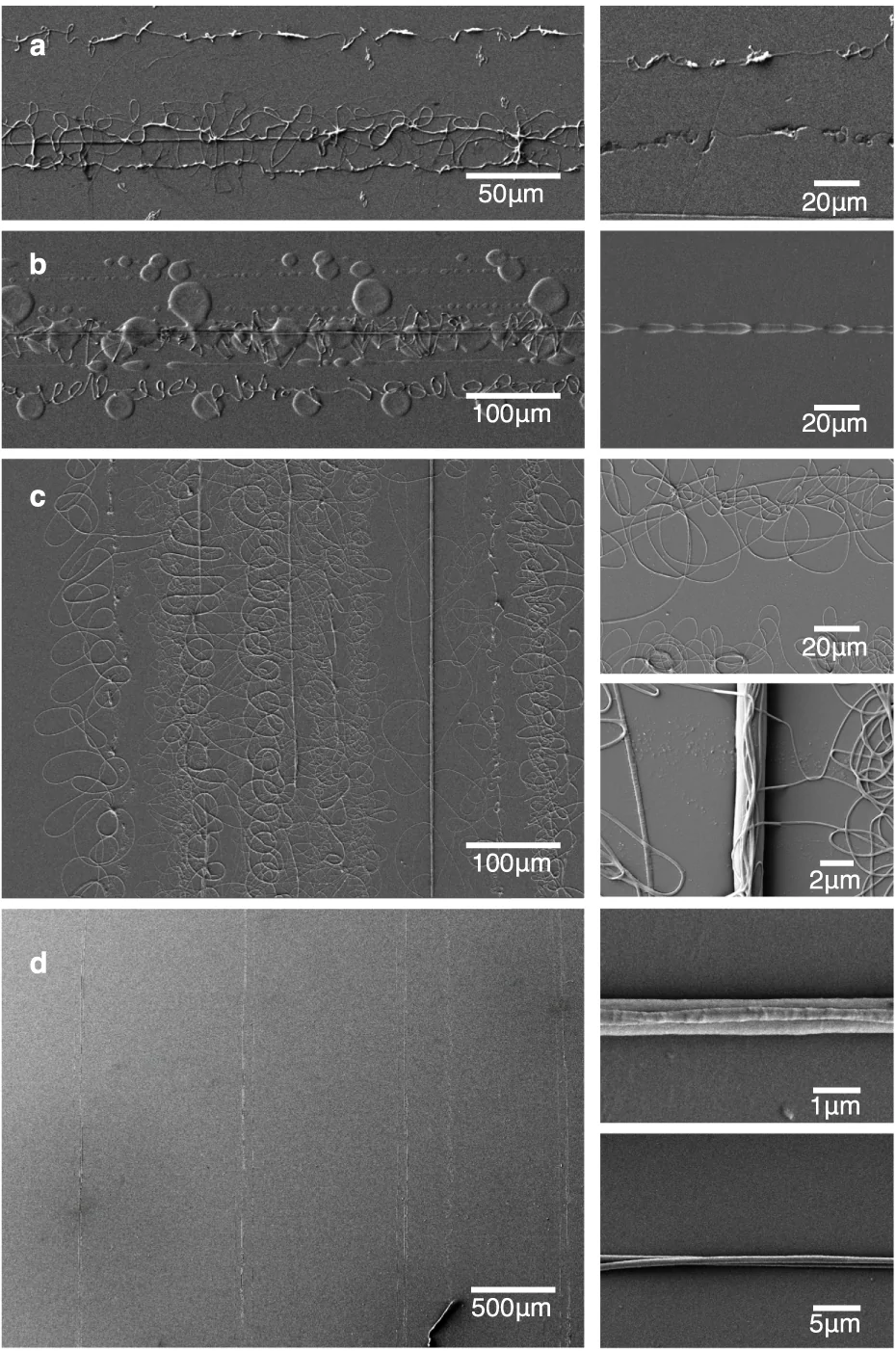
SEM images showing representative deposited morphologies at 2400 V under different flow-rate and collector-speed conditions. **(a)** Q=0.05 µL ${\text{min}}^{-1}$ and v=25 mm ${\text{s}}^{-1}$ ; **(b)** Q=0.3 µL ${\text{min}}^{-1}$ and v=25 mm ${\text{s}}^{-1}$ ; **(c)** At Q=0.1 µL ${\text{min}}^{-1}$ and v=25 mm ${\text{s}}^{-1}$ ; **(d)** At Q=0.1 µL ${\text{min}}^{-1}$ and v=75 mm ${\text{s}}^{-1}$ .

At the highest flow rate tested, Q=0.3 µL ${\text{min}}^{-1}$ , the deposited morphology became droplet-rich rather than fibre-like (Figure 6b). This result is consistent with the regime map, shown in Figure 4, where no stable cone-jet was observed at this flow rate. The supplied liquid volume appeared to have exceeded the ability of the electric field to stretch and remove material from the meniscus as a continuous jet. The excess flow therefore favoured meniscus swelling, droplet ejection, and local wet deposits instead of continuous fibre drawing.

At Q=0.1 µL ${\text{min}}^{-1}$ and v=25 mm ${\text{s}}^{-1}$ , continuous fibres with moderately good diameter uniformity were obtained (Figure 6c). However, the fibres were still curved and frequently coiled into ring-like features rather than forming straight tracks. This behaviour suggests that the jet arrival rate and collector translation were not fully matched. When the deposited jet length exceeds the distance removed by collector motion over the same time, compressive loading can develop in the freshly deposited filament, leading to local buckling, looping, or coiling (Fattahi et al., 2017; Qavi and Tan, 2021). Thus, although the cone-jet was sufficiently stable to produce continuous fibres, the collector speed was too low to fully stretch and place the filament as a straight line.

Increasing the collector speed to v=75 mm ${\text{s}}^{-1}$ at the same flow rate, Q=0.1 µL ${\text{min}}^{-1}$ , produced substantially straighter and more parallel fibres (Figure 6d). This condition provided a better balance between jet delivery, electrostatic stretching, and substrate translation. The inset shows local fibre-on-fibre contact or stacking, but such events were sporadic rather than systematically controlled. The result demonstrates that the transition from discontinuous, droplet-rich, or coiled deposits to straighter printed tracks was not controlled by flow rate alone, but by the combined deposition operating point, including collector speed. These observations are important for precision EHD fibre writing because they show that stable cone-jet formation is necessary but not sufficient for accurate line deposition; the substrate motion must also be tuned to the jet arrival rate.

#### 3.4.2 Fibre diameter under different electrohydrodynamic operating conditions

Fibre diameter was evaluated at a fixed flow rate of Q=0.1 µL ${\text{min}}^{-1}$ and v=75 mm ${\text{s}}^{-1}$ across practical operating conditions using 2000, 2,400, and 2800 V (Figure 7). The working distance was adjusted within the range 1.0–1.2 mm to maintain printing stability. Therefore, the resulting diameter trends are discussed in relation to the combined electrohydrodynamic operating point and nominal electric field, rather than voltage alone. Because the working distance was adjusted during printing to preserve a stable cone-jet, a single fixed value of E , d/h , or $Bo_{E}$ cannot be assigned to each voltage condition. Instead, the relevant operating ranges were E≈1.67 –2.00 kV ${\text{mm}}^{-1}$ and $Bo_{E}\approx 0.26$ –0.38 at 2000 V, E≈2.00 –2.40 kV ${\text{mm}}^{-1}$ and $Bo_{E}\approx 0.38$ –0.54 at 2400 V, and E≈2.33 –2.80 kV ${\text{mm}}^{-1}$ and $Bo_{E}\approx 0.51$ –0.74 at 2800 V; in all cases, d/h varied between approximately 0.42 and 0.50. The present dataset should not be interpreted as an independent voltage-sweep experiment at fixed geometry. These conditions fall within or close to the stable region identified in Figure 4, with the 2800 V condition approaching the upper electric-stress boundary of controlled deposition. For each condition, *N* represents the total number of individual fibre diameter measurements (at least 30) taken from calibrated SEM images acquired from different fields of view across three independently printed samples prepared on different days.

**FIGURE 7:**
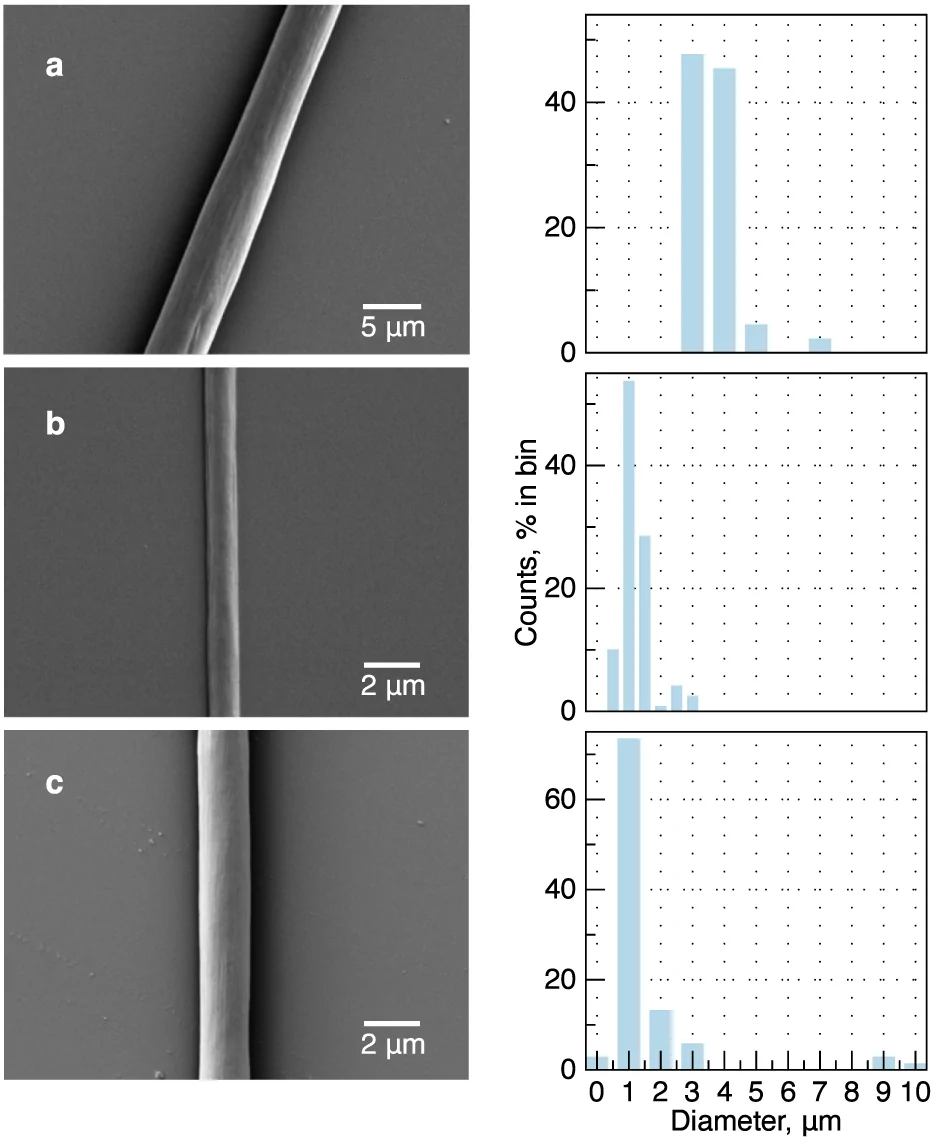
SEM images and corresponding fibre-diameter frequency distributions under different electrohydrodynamic operating conditions at Q=0.1 µL ${\text{min}}^{-1}$ , v=75 mm ${\text{s}}^{-1}$ , and a working distance of 1.0–1.2 mm. **(a)** 2000 V, **(b)** 2400 V, **(c)** 2800 V. The reported N values denote fibre-level diameter measurements from calibrated SEM images.

At 2000 V, fibres had a mean diameter of 3.85 ± 0.81 µm (N=30) . The SEM images showed smooth and continuous fibres, but the diameter distribution was broader than that observed at 2400 V. This condition is consistent with operation near the lower boundary of the stable window, where electric stress was sufficient to sustain a continuous jet but produced less jet thinning than at higher field strength.

At 2400 V, the mean fibre diameter decreased to 1.19 ± 0.49 µm (N=40) . This condition produced the narrowest distribution among the tested voltages and the most uniform low-micrometre fibres. The reduction in fibre diameter relative to 2000 V is consistent with stronger electrostatic stretching and jet thinning within the central region of the stable cone-jet window.

At 2800 V, the mean diameter was 1.53 ± 1.72 µm (N=31) . The standard deviation exceeded the mean, indicating a broad and highly skewed diameter distribution rather than a well-controlled normal population. The SEM image also showed fibres with visibly different diameters within the same deposit. This broad distribution is consistent with operation near the upper stability boundary, where intermittent transitions toward pulsating, displaced, or multi-jet behaviour can generate both thin jet-drawn fibres and thicker deposits. Therefore, the response at 2800 V is interpreted as the onset of instability rather than a reliable route for further diameter reduction.

#### 3.4.3 Patterning fidelity and spatial control

The spatial control of the EHD printing system was assessed using programmed snake-like parallel-line patterns (Figure 8a). At 2400 V, Q=0.1 µL ${\text{min}}^{-1}$ , and v=75 mm ${\text{s}}^{-1}$ , the printed pattern reproduced the millimetre-scale toolpath geometry, with parallel fibre tracks visible across the deposition area. Local accumulation of material was observed near turn points. This accumulation is attributed to increased dwell time during substrate deceleration and acceleration at trajectory reversals and could be reduced in future by stage-velocity optimisation together with voltage modulation, or temporary jet interruption at turns.

**FIGURE 8:**
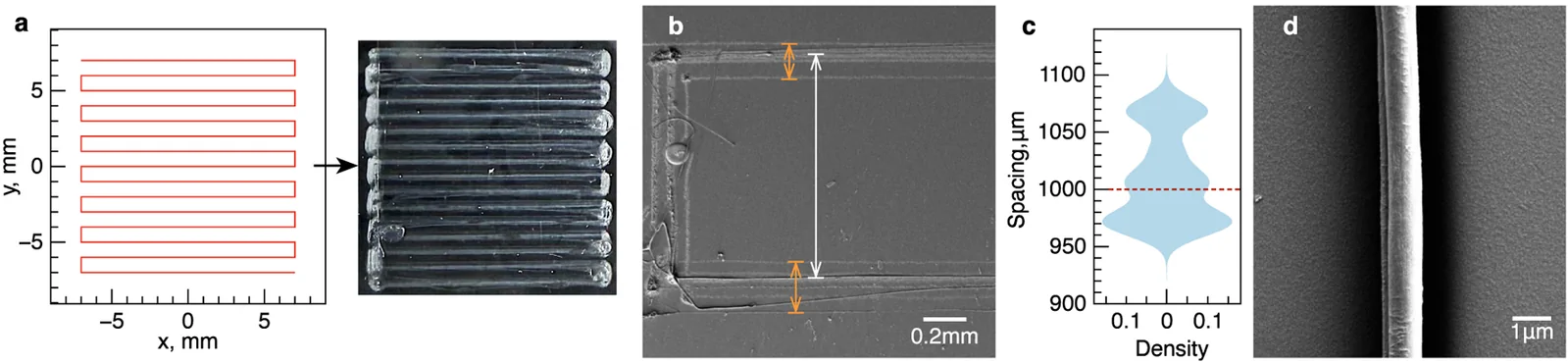
Programmed fibre deposition and spacing fidelity. **(a)** Programmed snake-like parallel-line toolpath and corresponding optical image of the printed fibre pattern obtained at 2400 V, Q=0.1 µL ${\text{min}}^{-1}$ , and v=75 mm ${\text{s}}^{-1}$ , showing reproduction of the target geometry. **(b)** Low-magnification SEM image of parallel printed fibres used to measure line spacing. The white arrow indicates an example spacing measurement, while orange arrows indicate local variation artefacts. This pattern was produced using four repeated passes at v=75 mm ${\text{s}}^{-1}$ , corresponding to four deposited fibre “layers” or sequential fibres along each programmed line. **(c)** Violin plot of measured line spacing for the fibres shown in panel **(b)**, giving a mean spacing of 1,010 ± 41 µm against a programmed value of 1,000 μm, indicated by the red dotted line. The mean absolute spacing error was 3.3% ( N=12 measurements across three imaging fields). **(d)** High-magnification SEM image showing local fibre-on-fibre stacking, where one fibre is deposited above another. Such stacking demonstrates the possibility of vertical fibre placement, but occurred only sporadically with the current open-loop setup.

Inter-fibre spacing was measured from low-magnification SEM images of the patterned deposits (Figure 8b,c). For the pattern shown in Figure 8b, four repeated passes were printed along the programmed path at v=75 mm ${\text{s}}^{-1}$ , corresponding to four deposited fibre “layers” or sequential fibres at each line location. The programmed spacing was 1,000 μm, while the measured spacing was 1,010 ± 41 μm, with a mean absolute spacing error of 3.3% ( N=12 measurements across three imaging fields). These values indicate millimetre-scale pattern fidelity, with the spacing analysis representing within-pattern fidelity for the analysed printed structure. Nevertheless, the spacing variation remains large relative to the fibre diameter of 1.19 ± 0.49 μm at the same operating condition. The positional error should therefore be considered a current limitation of the open-loop printing system rather than evidence of micrometre-scale placement accuracy.

High-magnification SEM imaging showed occasional locations where one fibre was deposited directly on top of another (Figure 8d), with a similar local stacking also visible in the inset in Figure 6d. These observations demonstrate that fibre-on-fibre placement and local vertical stacking are physically possible with the present EHD printing platform. However, such events occurred only sporadically and were not yet consistent across the four-pass patterns. The present system therefore demonstrates controlled two-dimensional fibre writing with occasional local 3D stacking, but not reliable layer-by-layer three-dimensional fibre printing.

The observed spacing variability and rare stacking events are likely caused by stochastic jet perturbations, local electrostatic interactions with previously deposited fibres, residual charge on the substrate or fibre surface, and mechanical vibration of the stage and needle. More deterministic 3D fibre assembly will require improved control of the jet landing position relative to previously deposited material, as well as closed-loop synchronisation between collector motion, voltage modulation, flow rate and stage movement.

Although controlled fibre-on-fibre stacking was not achieved, the occasional local stacking events identify a feasible route toward future 3D EHD fibre assembly.

## 4 Discussion

This study establishes a process-structure approach for near-field EHD printing of an aqueous gelatin-GPTMS-PEO hybrid biomaterial ink. Acetic acid acted as a process-modifying additive that improved apparent precursor homogeneity, delayed the cooling-induced viscosity increase, increased conductivity, and reduced surface tension, thus providing stable cone-jet formation. Since the present study focused on the selected 6% v/v formulation, systematic acid-concentration screening remains a route for future optimisation. FTIR-ATR and Raman spectroscopy supported retention of the principal gelatin and PEO vibrational signatures after acidification and printing. Mapping processing parameters in terms of electric Bond number $Bo_{E}$ , capillary number Ca , and d/h showed that fibre formation required simultaneous control of electric stress, ink flow rate, and needle-collector geometry rather than voltage alone. The morphology and patterning results show that the GG-PEO precursor ink can be printed as continuous micrometre fibres when operated within the stable cone-jet window around 2400 V, Q=0.1 µL ${\text{min}}^{-1}$ , and v=75 mm ${\text{s}}^{-1}$ . Programmed parallel patterns were printed with a mean spacing of 1,010 ± 41 µm against a nominal 1,000 μm spacing, demonstrating millimetre-scale programmed fibre deposition and establishing a practical basis for future ordered fibrous architectures that could be evaluated for biointerface-relevant or scaffold-mimetic applications. Additionally, the morphology data indicate that both flow rate and collector speed v are critical parameters. At Q=0.05 µL ${\text{min}}^{-1}$ , the deposits followed the toolpath but were discontinuous, whereas Q=0.3 µL ${\text{min}}^{-1}$ produced droplet-rich deposits because the liquid supply exceeded stable electrical stretching. At Q=0.1 µL ${\text{min}}^{-1}$ , v=25 mm ${\text{s}}^{-1}$ produced continuous fibres but retained coiling and ring-like features, while increasing the collector speed to v=75 mm ${\text{s}}^{-1}$ produced straighter parallel tracks. This suggests that future process optimisation should focus on matching collector velocity to jet deposition speed.

Future development of this EHD platform will therefore focus on improving positional control during starts, stops, turns, and repeated passes. Stage acceleration smoothing and trajectory-dependent velocity control should reduce material accumulation at snake-pattern turn points. Dynamic voltage or flow-rate modulation during toolpath reversals could further suppress local over-deposition while maintaining a stable meniscus. Environmental enclosure with controlled humidity is expected to reduce air-current-induced jet perturbations and improve control over solvent evaporation, fibre drying, and charge dissipation, while improved charge management, including controlled charge dissipation intervals, or active neutralisation between passes, should reduce electrostatic deflection by previously deposited fibres. For application-specific development, biological performance, residual acidity, aqueous stability, swelling, degradation, and mechanical properties of the printed fibres will also need to be evaluated directly. Finally, real-time optical tracking of the meniscus and jet landing position could provide closed-loop correction of lateral drift. This capability could be strengthened by incorporating current monitoring and current-based feedback through the Keithley high-voltage supply, since the electrical current provides a direct process signal linked to charge transport and jet stability. In future, the system could be developed into an adaptive, self-optimising EHD printing platform in which machine-learning algorithms analyse the real-time camera feed, classify meniscus shape and jetting state, and automatically adjust voltage, flow rate, working distance, collector speed, temperature, or humidity to maintain consistent fibre jetting. These developments form the next step toward micrometre-scale placement accuracy and reproducible layer-on-layer EHD fibre assembly, building directly on the occasional fibre-on-fibre stacking events observed in the present study.

## Data Availability

The raw data supporting the conclusions of this article will be made available by the authors, without undue reservation.
